# Evaluating efficacy of indoor non-pharmaceutical interventions against COVID-19 outbreaks with a coupled spatial-SIR agent-based simulation framework

**DOI:** 10.1038/s41598-022-09942-y

**Published:** 2022-04-13

**Authors:** Chathika Gunaratne, Rene Reyes, Erik Hemberg, Una-May O’Reilly

**Affiliations:** 1 Massachusetts Institute of Technology, Computer Science and Artificial Intelligence Laboratory, Cambridge, MA USA; 2 Present Address: Oak Ridge National Laboratory, Oak Ridge, TN USA

**Keywords:** Computational models, Respiratory tract diseases

## Abstract

Contagious respiratory diseases, such as COVID-19, depend on sufficiently prolonged exposures for the successful transmission of the underlying pathogen. It is important that organizations evaluate the efficacy of non-pharmaceutical interventions aimed at mitigating viral transmission among their personnel. We have developed a operational risk assessment simulation framework that couples a spatial agent-based model of movement with an agent-based SIR model to assess the relative risks of different intervention strategies. By applying our model on MIT’s Stata center, we assess the impacts of three possible dimensions of intervention: one-way vs unrestricted movement, population size allowed onsite, and frequency of leaving designated work location for breaks. We find that there is no significant impact made by one-way movement restrictions over unrestricted movement. Instead, we find that reducing the frequency at which individuals leave their workstations combined with lowering the number of individuals admitted below the current recommendations lowers the likelihood of highly connected individuals within the contact networks that emerge, which in turn lowers the overall risk of infection. We discover three classes of possible interventions based on their epidemiological effects. By assuming a direct relationship between data on secondary attack rates and transmissibility in the agent-based SIR model, we compare relative infection risk of four respiratory illnesses, MERS, SARS, COVID-19, and Measles, within the simulated area, and recommend appropriate intervention guidelines.

## Introduction

Establishing safe return-to-work guidelines is essential to avoiding COVID-19 outbreaks. Respiratory diseases such as COVID-19 are often spread through droplet or aerosol transmission of the virus^1–4^, where spatial proximity and duration of closeness are important factors towards the transmission of pathogens. Non-pharmaceutical interventions have been shown to be crucial in the effort to reduce spread of COVID-19 and must be implemented alongside vaccinations to reduce the number of hospitalizations and deaths^5,6^. Therefore, it is vital that organizations properly assesses the spatial effects of imposed non-pharmaceutical interventions on their ability to reduce risk of infection among the population by suppressing prolonged contacts. Floor layouts, walls, hallways, and other physical obstacles, in addition to safety guidelines such as recommended break duration, may restrict certain contacts from occurring while amplifying others, and spatial agent-based models are able to simulate these factors. Modeling these spatial effects provides a more accurate representation of the plausible patterns of prolonged exposures that may occur within a workplace and help in the generation of contact networks that can then be studied regarding their resilience to disease outbreaks.

In the current context of increasing interest and resources toward computational research, the COVID-19 pandemic has generated great interest in mathematical modeling of infectious diseases aimed at projecting case counts and mortality, vaccination efficacy, efficacy of non-pharmaceutical interventions, and economical impacts^7–11^. Common approaches reported in the literature for modeling the spread of infectious disease include multi-compartmental models, contact network models, and agent-based models (also referred to as individual-based models)^12–14^. While multi-compartmental models have had great success in forecasting progression of contagion, they are based on the assumption of full and homogenous mixing among the population. In reality, contact behavior is crucial to understanding the spread of disease and is determined through a multitude of social, cultural, political, economic, and behavioral factors that vary over the study cohort^15^. In contrast, network-based or agent-based approaches have the ability to simulate individual-scale behaviors and the resulting shifts in local contact patterns, which in turn scale superlinearly with population size. Agent-based models are ideal at replicating real-world spatial movement patterns and have been used to assess the spread of infectious diseases such as COVID-19 in both indoor and outdoor settings^9,10,15–19^. For instance, Epstein et al., demonstrated the significant impact on overall spatio-temporal dynamics of disease spread caused by fear-related responses of self-isoloation and flight, highlighting the importance of modeling such behaviors when projecting future case counts^20^. A survey of mathematical modeling of infectious diseases in China found that individual-based modeling rose in popularity since 2003 to study a variety of disease including SARS in China^21^. Childs et al, found that out of 24 articles on mathematical models of disease spread, a majority modeled viral pathogens among human hosts^22^, with agent-based modeling of between-host dynamics being used in some studies and transmission rates commonly used as a linking mechanism between hosts. Another systematic literature review of 210 articles modeling transmission of environmentally persistent zoonotic diseases finds that roughly 12% of articles were found to employ agent-based models^23^.

Numerous studies have utilized agent-based models to investigate the impact of non-pharmaceutical interventions including self-isolation, quarantine, and social distancing on the spread of COVID-19^6,24–28^. Agent-based models have been more commonly utilized to investigate scenarios where the effectiveness of physical, non-pharmaceutical interventions within a localized setting, such as within a hospital, were of interest^29^. In an analysis of 698 research articles on agent-based modeling of infectious disease transmission, from 2006 to 2015, Willem et al find that 261 studies simulated interventions, out of which 105 studies investigated non-pharmaceutical interventions, in particular^30^. Furthermore, the full text analysis of 24 studies focused on vaccine-preventable childhood diseases, excluding influenza, revealed the successful use of the AnyLogic simulation platform for simulating up to 100000 people over periods of up to 2000 days^30^. Studies have shown that the presence of highly connected individuals, or hubs, in social networks, is a leading factor, facilitating large outbreaks among populations^31^. While many agent-based approaches report a lack of micro-scale data for the re-construction of actual contact networks and opt to use theory-based, social network generation algorithms^28^, others overcome this challenge by modeling pedestrian dynamics with randomized, full mixing^26,32^, or by using schedule and mobility information to generate patterns of contact for greater simulation fidelity^1,6,33,34^, as we have done in this study.

We demonstrate how spatial agent-based modeling can be used to predetermine contact networks under varying intervention strategies, under constraints specified by the Centers for Disease Control (CDC) definition of a *prolonged contact* for SARS-CoV-2 transmission^35^. We generated contact networks using a spatial-agent based model implemented in AnyLogic, which incorporates room and corridor locations, entrances and exits, arrival schedules, restroom locations, and break areas. We investigate the effects of non-pharmaceutical intervention strategies based on three dimensions: one-way or unrestricted movement, population size, and frequency of leaving one’s designated office for breaks. The generated networks were then subjected to an agent-based SIR model to compare the resilience of the simulated intervention strategies against viruses with different transmission rates. Finally, we used known secondary attack rates for four respiratory illnesses, MERS, SARS, COVID-19 (Delta and prior variants), and Measles, in order to approximate final infection ratios expected for each disease under the simulated intervention strategies.

## Methodology

**Figure 1:**
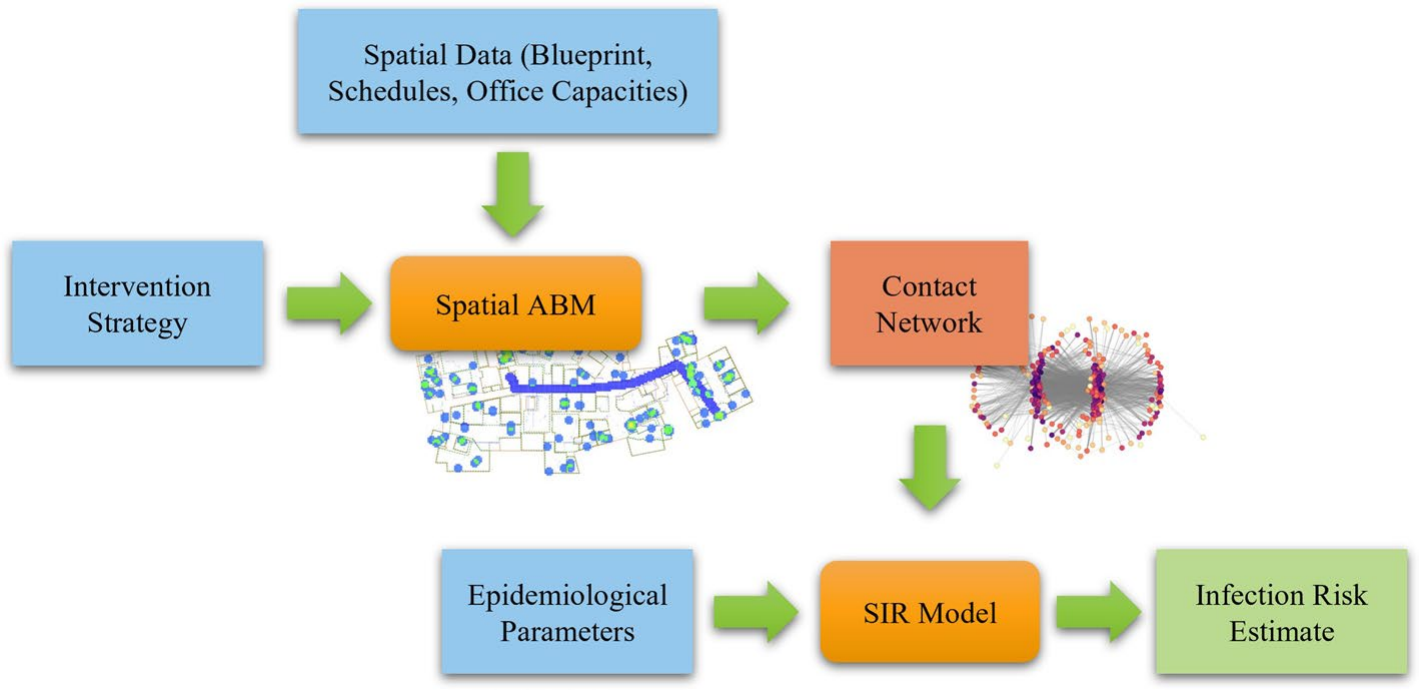
System diagram.

We coupled two agent-based models to assess operational efficacy of COVID-19 intervention strategies on the Stata center at Massachusetts Institute of Technology, by assessing resulting risks of infection. First, as shown in Fig. 1, a data-informed, spatial agent-based model was used to simulate individual movement within a floor of the building under varying physical intervention constraints, to generate high-risk contact networks. Second, an agent-based SIR model was simulated on the generated contact networks to obtain final infection ratios of the population.

### Spatial agent-based model

The spatial agent-based model was implemented to mimic the movement of personnel under restrictions imposed by varying constraints of the intervention strategies. The model was implemented in the AnyLogic simulation software^36^, which allowed for accurate representation of physical spaces including offices, stairwells, and restrooms within the simulated area. The second floor of the Stata center was used for this purpose, which hosts several CSAIL research offices and labs, conference rooms, rest areas, two restrooms, utility space, and pantry area. The blueprint image of the building floor under consideration was loaded into AnyLogic and AnyLogic’s *wall* construct from the *pedestrian library* was used to designate areas impassable by agents, forming office spaces and rooms. Stairwells and elevators were identified from the blueprints and *targetlines* from the *pedestrian library* were allocated at these points within the model from which agents could enter or exit the floor. Office spaces were demarcated with their real-world identifiers and assigned capacities according to the current office capacities set by building administration.

**Figure 2:**
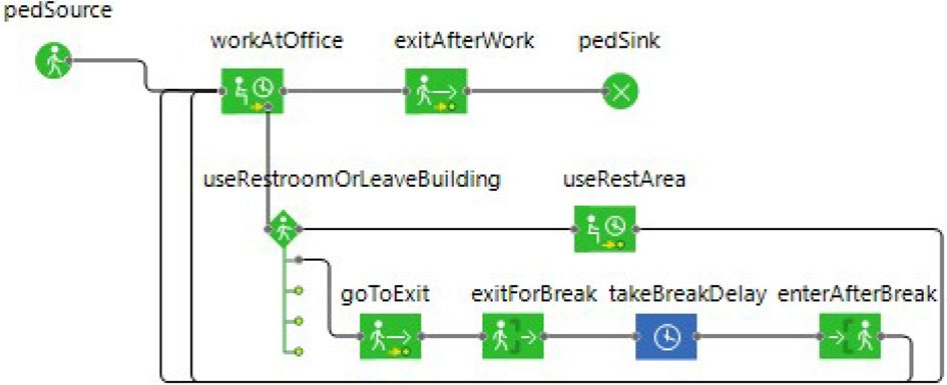
State machine driving agent behavior within the simulated environment of the spatial agent-based model, implemented using AnyLogic’s Pedestrian library.

Agent behavior was controlled using a state machine implemented with components from AnyLogic’s *pedestrian library* as shown in Fig. 2. Individuals were generated from a pedestrian source, at which they were randomly assigned to an available office. We used an approximation of a daily schedule for the floors as shown in Table 1. Individuals began entering the floor from around 6am at a very low rate, followed by a gradual increase in rate towards 9am, ending with a gradual decrease in rate towards 11am. We assumed that individuals would work a complete 8 h shift after which they would exit the floor. This meant that departure times would follow a symmetrical distribution to that of entrance times. The agent would then enter the simulation from a designated stairwell or elevator according to the intervention strategy’s movement restriction as detailed below. Once on the floor, the agent would move towards its office location along the hallways created by the wall objects. Collision detection and path-finding algorithms were handled by AnyLogic and comfortable walking speed was selected for each individual from a uniform distribution between 0.5 and 1 meters per second. Once at the office, individuals remained at a chosen location within the office space, unless they were interrupted to take a break at an hourly probability of $\alpha$. There was a 0.5 probability that they would remain on the floor in designated break locations and a 0.5 probability that they would exit the floor during this time. Break locations included the restrooms and common areas on the floor and the time agents would spend at a break location followed a uniform distribution between 5 and 20 min. Individuals that exit the floor during their break would use designated exits and entrances according to the movement restriction in place and the time spent outside was chosen from a triangular distribution of minimum, maximum, and mode of 20, 60, and 30 min, respectively.

**Table 1:** Schedule used for controlling agent entrance to the floor over time.

Start time	End time	Proportion of population
6:00am	7:00am	0.05
7:00am	8:00am	0.15
8:00am	9:00am	0.4
9:00am	10:00am	0.3
10:00am	11:00am	0.1

Each hour, the specified proportion of the population enters the floor from the designated entrances and heads to their respective office spaces. Agents spend 8 h within the simulation and then exit for the day, implicitly making the exit schedule symmetrically distributed.

**Figure 3:**
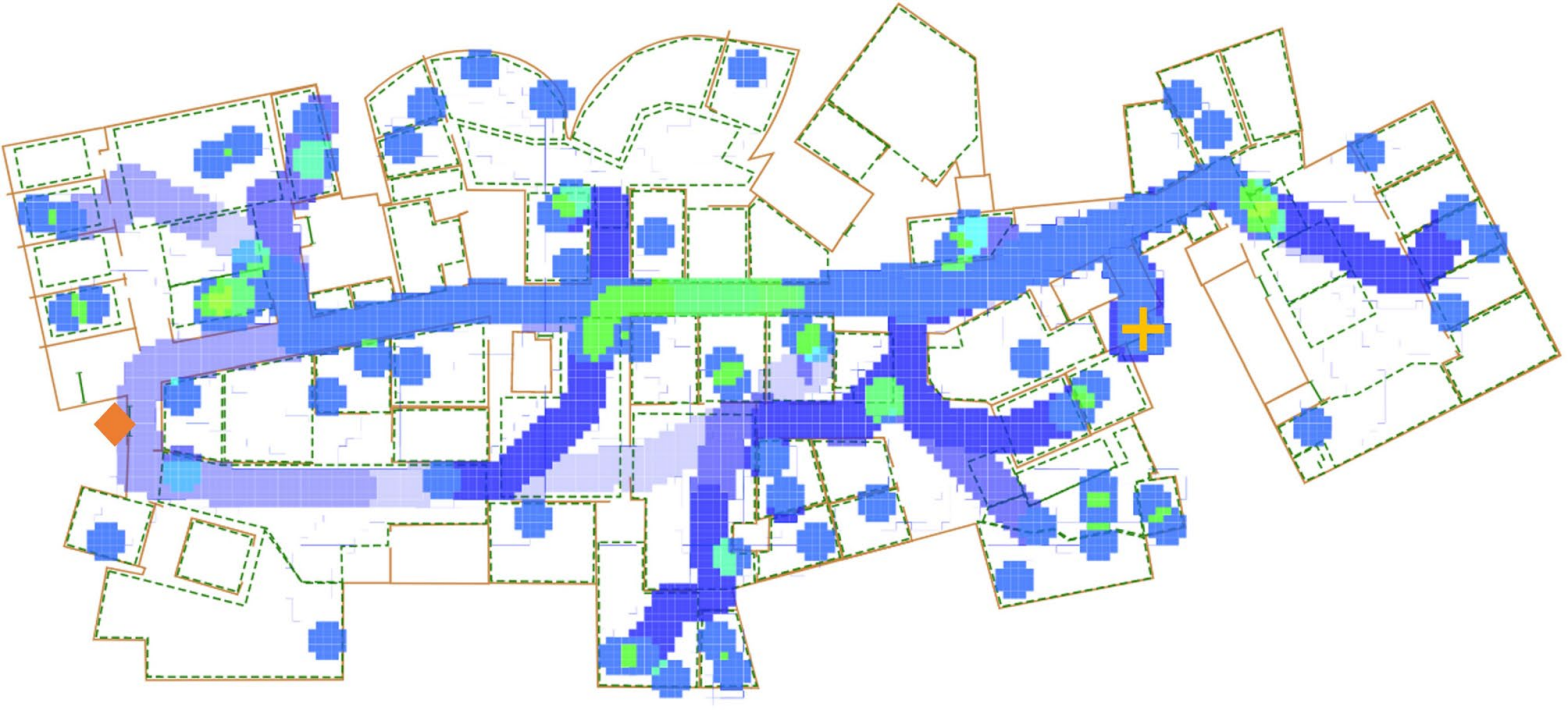
Example run of the spatial agent-based model with one-way movement restriction enabled. A heatmap depicting agent movement through the simulated floor is overlayed (lighter colors showing where agents have recently spent more time at). The designated entrance is marked with a yellow plus symbol and the designated exist is marked with an orange diamond symbol. In unrestricted movement, both these locations can be used for both entrance and exit to the space.

Intervention strategies were decomposed into three dimensions, namely movement restriction, population capacity, hourly break probability. Two movement restrictions were simulated: (1) unrestricted movement, where individuals were able to enter and exit the floor from any of the available stairwells and elevators, and (2) one-way movement, where entrance and exits to the floor were only allowed at separate, designated locations as shown in the map in Fig. 3. Population capacities were controlled using a population multiplier parameter, $\beta$, applied to the recommended capacities of each office. For each office, *o*, the population capacity, $n_o$ was calculated as $n_o = \beta c_o$, where $c_o$ was the capacity for *o* recommended by the administration. Thus the total population for the simulation was $\sum _{o \in O}{\beta c_o}$, where *O* is the complete collection of offices. Hourly probability of taking a break, $\alpha$, determined whether agents would leave their offices for a break as described above and utilize the break locations or exit the floor temporarily, controlling the rate at which agents may contact one another in the hallways or common areas. $\beta$ was varied in the range [0.25, 2.0] in increments of 0.25 and $\alpha$ was varied in the range [0.05, 0.45] in increments of 0.05, and along with the two forms of movement restriction resulted in 128 separate strategies. Each strategy simulation was replicated 10 times to account for stochasticity.

Contacts leading to high infection risk were measured from simulations of the spatial agent-based model by considering the CDC definition of a prolonged contact between an infected individual and a susceptible individual, which would lead to a high risk of transmission of the SARS-CoV-2 virus^35^. According to CDC guidelines at the time of writing, a prolonged contact occurs when a susceptible individual and infected individual come within 6 feet of one another for over a 15 min duration (note: We have not accounted for changes in these parameters under varying degrees of PPE usage.). We used this definition to track agents in our model that came into prolonged contact by recording instances in the spatial agent-based model simulations when two agents spend 15 min or more within 6 feet of one another. These contacts were then treated as graph edges and used to construct an undirected prolonged contact network for each simulation.

### Epidemic simulation on prolonged contact networks

An agent-based SIR model was used to simulate the viral spread over the contact networks generated from the spatial agent-based model. The goal of the agent-based SIR model was to assess the infection risk posed by the intervention strategies that generated the contact networks when a single infected individual would arrive during a workday. The agent-based SIR model was executed as follows on each contact network. Each agent $m \in M$ of the population of agents *M* represented an individual person. The population of agents was initialized such that an agent, *m*, was created for each node in the contact network being simulated. The other agents that *m* would interact with over the entire simulation period were those agents, $N_m \in M$, who represented the neighboring nodes of the node represented by *m* in the contact network.

We assume a homogeneous parameter $\rho$ to represent the transmissability of the virus, where $\rho$ equals the proportion of susceptible neighbors of an infected agent that will become infected as well. Say, that agent’s infection state is represented by the function $\theta : M -> \{S,I,R\}$, where $\{S,I,R\}$ are the susceptible, infectious, and recovered states that any *m* may exist in. All agents but one were set to a susceptible state, $\theta (m_s)=S \mid m_s \in M$, while a single, randomly selected agent, $m_i \in M$, was chosen and set to an infectious state, $\theta (m_i)=I$. A proportion, $\rho$, of the neighbors of *m*, $\rho (N_m) \mid \rho (N_m) \subset N_m$, was then randomly chosen and changed to infectious, $\theta (\rho (N_m))=I$. The infecting agent was then changed to a recovered state, $\theta (m_i)=R$, and could no longer infect other agents. The process was then repeated for the newly infected agents and continued until no further infected agents remained, $\theta (M) \equiv I = \emptyset$. Each contact network and $\rho$ configuration, was replicated 10 times to account for stochasticity and heterogeneity in node degree centrality. The final infected ratio of the entire population, $\phi$, was measured from each agent-based SIR simulation as the proportion of recovered agents, $\Phi \mid \Phi = \theta (M) \equiv R$, at the end of the simulation:

1 $$\begin{aligned} \phi = \frac{|\Phi |}{\sum _{o \in O}{\beta c_o}} \end{aligned}$$

Finally, we use secondary attack rate (SAR) in order to assess the efficacy of the simulated interventions at minimizing $\phi$ for the respiratory diseases, MERS, SARS, COVID-19, and Measles. SAR, also known as secondary infection risk, is an epidemiological measurement of the proportion of susceptible individuals that are infected due to close contact with an infected individual^37^. SAR is especially useful for quantifying *household infectivness* as it considers contacts that occur among individuals within close confines, and is applicable to our study, where individuals spend most of their workday in the same shared space. Assuming SAR to be a good approximation of $\rho$ in our model, we use empirically estimated 95% confidence intervals of mean SAR for four respiratory diseases from the literature to define ranges of $\rho$ as follows, MERS: [0.009, 0.107]^37^, SARS [0.048, 0.107]^37^, Measles [0.520, 0.846]^37^, earlier variants of COVID-19: [0.14, 0.22]^38,39^, and the more contagious Delta variant: [0.20, 0.32] among unvaccinated individuals^38–40^. At the time of writing, there was no peer-reviewed study estimating the SAR of the COVID-19 Omicron variant to our knowledge that would could include in our analysis. Thus, by running the agent-based SIR model with $\rho$ within the respective range for the simulated disease, we are able to provide predicted final infection ratios for each disease under the simulated intervention strategies.

## Results

This section presents the simulation results with respect to the three intervention parameters:

- Movement restriction; Either restricted to one-way movement or unrestricted movement across the floor.
- The hourly likelihood of taking a break, $\alpha$.
- The population multiplier applied to recommended population capacity, $\beta$.

The effects on maximum degree centrality, $C_{max}$, and proportion of agents with at least one contact, *K*, of the generated contact networks, alongside final infected ratios, $\phi$, under varying transmissibility, $\rho$, on these networks were observed.

**Figure 4:**
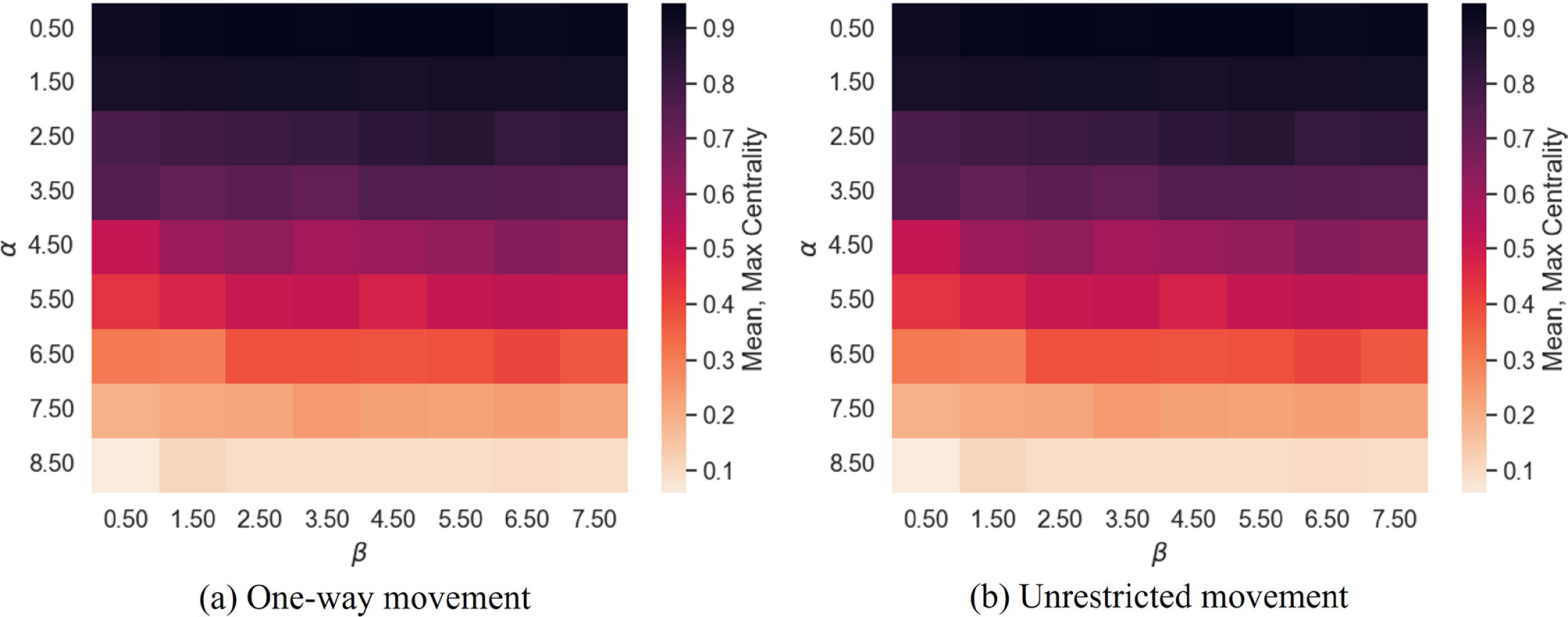
Mean maximum centrality, $\overline{C_{\text {max}}}$, of contact networks generated under varying $\alpha$, $\beta$, and form of movement restriction.

Figure 4 displays, $\overline{C_{\text {max}}}$, the mean of maximum degree centralities of the contact networks generated by the spatial agent-based model for each intervention strategy. $\alpha$ has the greatest effect on $\overline{C_{\text {max}}}$, with $\alpha =0.45$ being sufficient to produce at least one *hub* that was connected to approximately the entire network. There is no change in $\overline{C_{\text {max}}}$ with movement restriction (Fig. 4). $\alpha$ is seen to have a significantly positive correlation with $\overline{C_{\text {max}}}$ (Spearman rank correlation: $r_s=0.8674$, $p~{\text {value}}<2.673\times 10^{-194}$) and a significant, yet weaker, positive correlation is seen between $\beta$ and $\overline{C_{\text {max}}}$ (Spearman rank correlation: $r_s=0.08771$, $p~{\text {value}}=1.7781\times 10^{-195}$).

**Figure 5:**
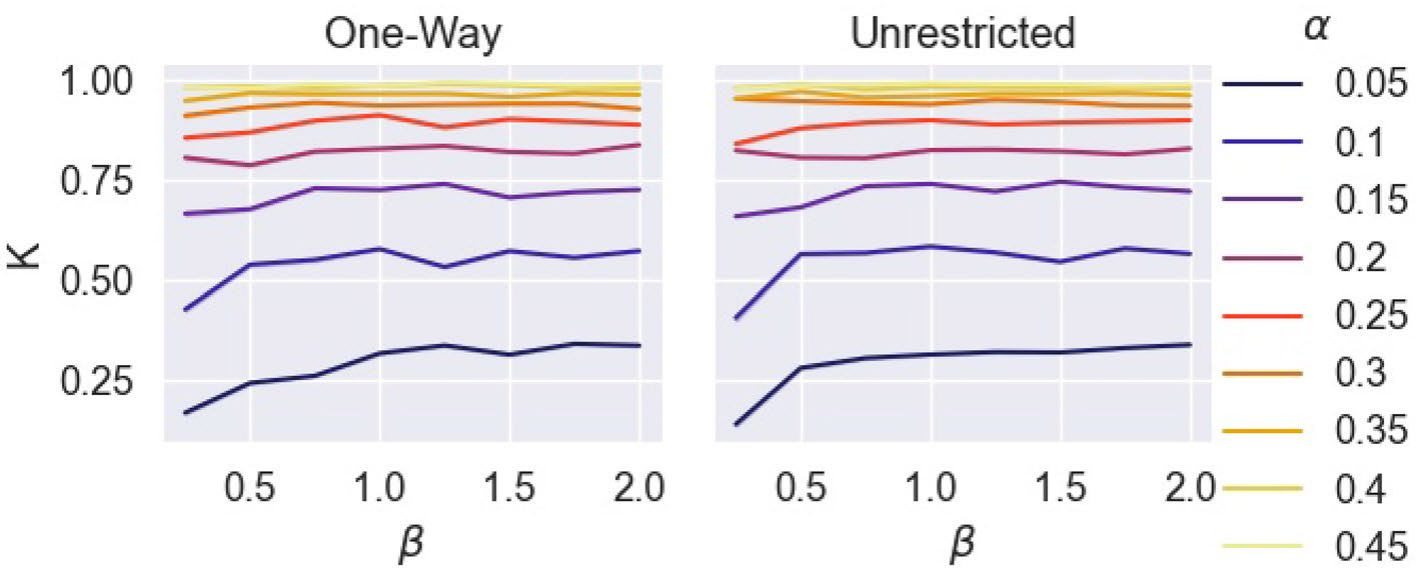
Proportion of agents with at least one prolonged contact *K* under varying $\alpha$, $\beta$, and form of movement restriction.

Figure 5 displays the proportion of agents in each simulation with at least one contact, *K*, against the three dimensions of intervention. Since the total population of each simulation was known, *K* was calculated as:

2 $$\begin{aligned} K =&\frac{|M|}{\sum _{o \in O}{\beta c_o}} \end{aligned}$$

Again, it is seen that $\alpha$ has the strongest effect on *K*, (Spearman rank correlation: $r=0.8952$, $p~{\text {value}}<2.673\times 10^{-194}$). When $\alpha > 0.1$, at least more than half the population was expected to have at least one prolonged contact regardless of the other two parameters. $\beta$ had a slight positive correlation with *K* (Spearman rank correlation: $r=0.0989$, $p~{\text {value}}<2.673\times 10^{-194}$) , which diminished at higher values of $\alpha$. Once more, the two forms of movement have restriction have no apparent effect on *K*.

**Figure 6:**
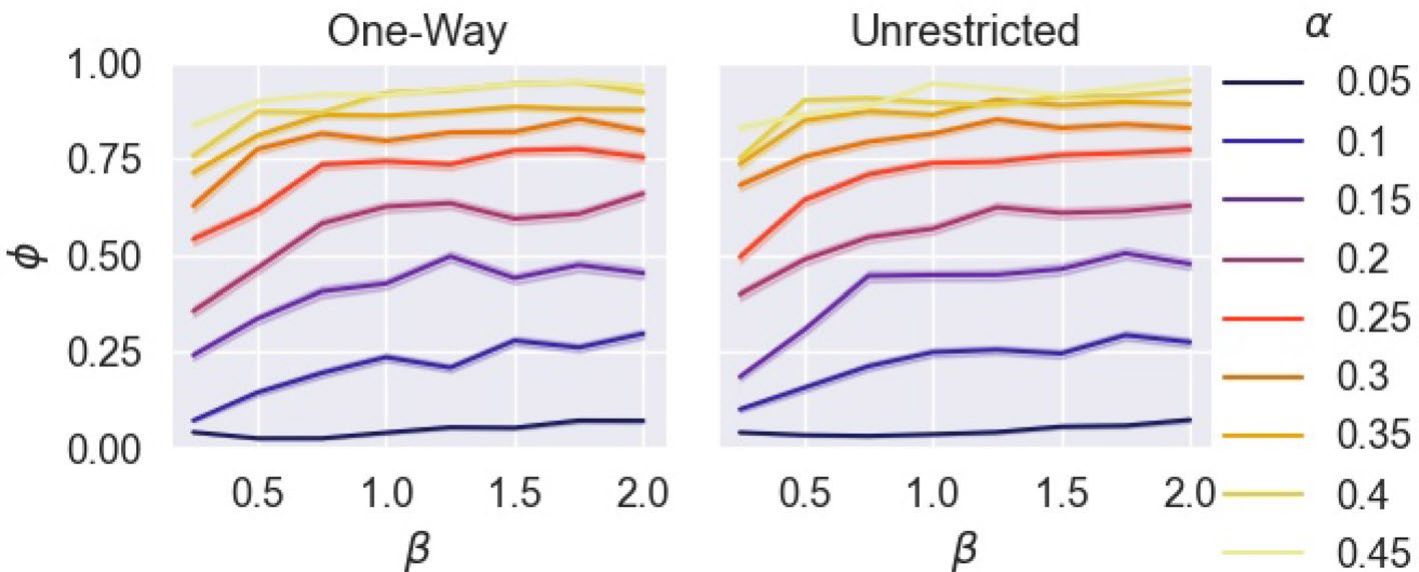
Final ratio infected by the number of times larger the population is by recommended capacity, by hourly break probability, for one-way and unrestricted movement.

Figure 6, displays outbreak size as final infected ratio, $\phi$, by $\beta$ and $\alpha$ for both forms of movement. Both forms of movement show similar behaviors under varying $\alpha$ and $\beta$. There is a significant positive correlation between $\alpha$ and $\phi$ (Spearman rank correlation: $r = 0.7954$, $p~{\text {value}}<2.673\times 10^{-194}$) and a weaker positive correlation between $\beta$ and $\phi$ (Spearman rank correlation $r=0.1916$, $p~{\text {value}}<2.673\times 10^{-194}$). At the recommended population capacity ($\beta = 1$), $\phi$ can be maintained under 0.5, if $\alpha < 0.1$, while more than half the population is at risk when $\alpha > 0.1$. At $\alpha < 0.1$, it is possible to keep at least half the population uninfected even when double the recommended population capacity is present, i.e. $\beta =2$.

**Figure 7:**
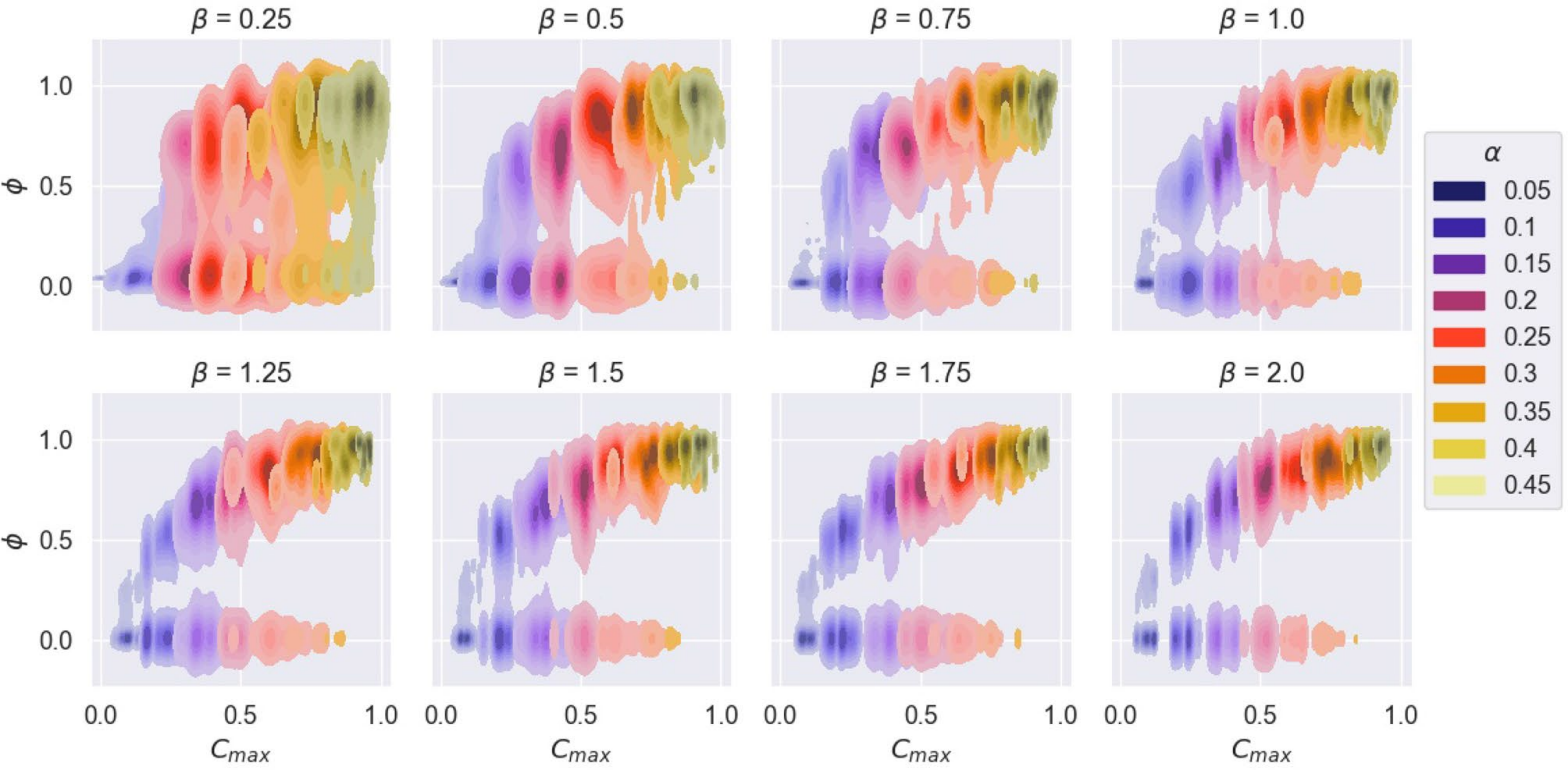
Density plot of $\phi$ by maximal network centrality, $C_\text {max}$, under varying $\alpha$ and $\beta$.

Figure 7 displays the relationship between maximum degree centrality per contact network, $C_{\text {max}}$, and $\phi$, for varying $\alpha$ and $\beta$. The correlation between $\alpha$ and $C_{\text {max}}$ is intensified with increasing $\beta$. At high $\beta$, a non-linear relationship between $C_{\text {max}}$ and $\phi$ can be seen. In other words, for sufficiently large population sizes, higher hourly break probability can cause networks with higher maximum centrality, leading to larger networks with highly connected hubs that allow for higher final infection ratios.

**Figure 8:**
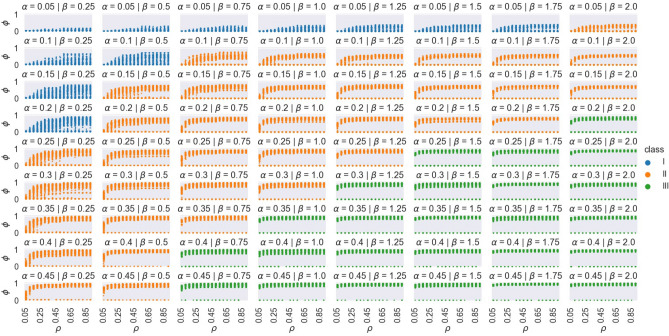
The effect of $\rho$ on $\phi$ under different $\alpha$ and $\beta$. Three classes of behavior can be seen.

Figure 8, shows the effects of $\rho$ on $\phi$ under different $\alpha$ and $\beta$ conditions. Under varying $\alpha$ and $\beta$, the effect that $\rho$ has on $\phi$ can be separated into thee classes. Class I (blue) where gradually larger ranges of $\phi$ can be expected with higher $\rho$, but $\phi$ stays generally less than 1; Class II (orange) where there is an initial gradual increase in possible range of $\phi$ with $\rho$, followed by a bifurcation into an oscillation of period 2, i.e. either very low $\phi$ or higher ranges of $\phi$ ($> 0.5$), which eventually transitions into $\phi \approx 1$ for $\rho > 0.5$; and Class III (green) where the final states exist in an oscillation of period 2 for $\rho <= 0.5$, at $\phi > 0.75$ and $\phi \approx 0$, and for $\rho > 0.5$ a steady state of $\phi > 0.75$ is observed.

**Figure 9:**
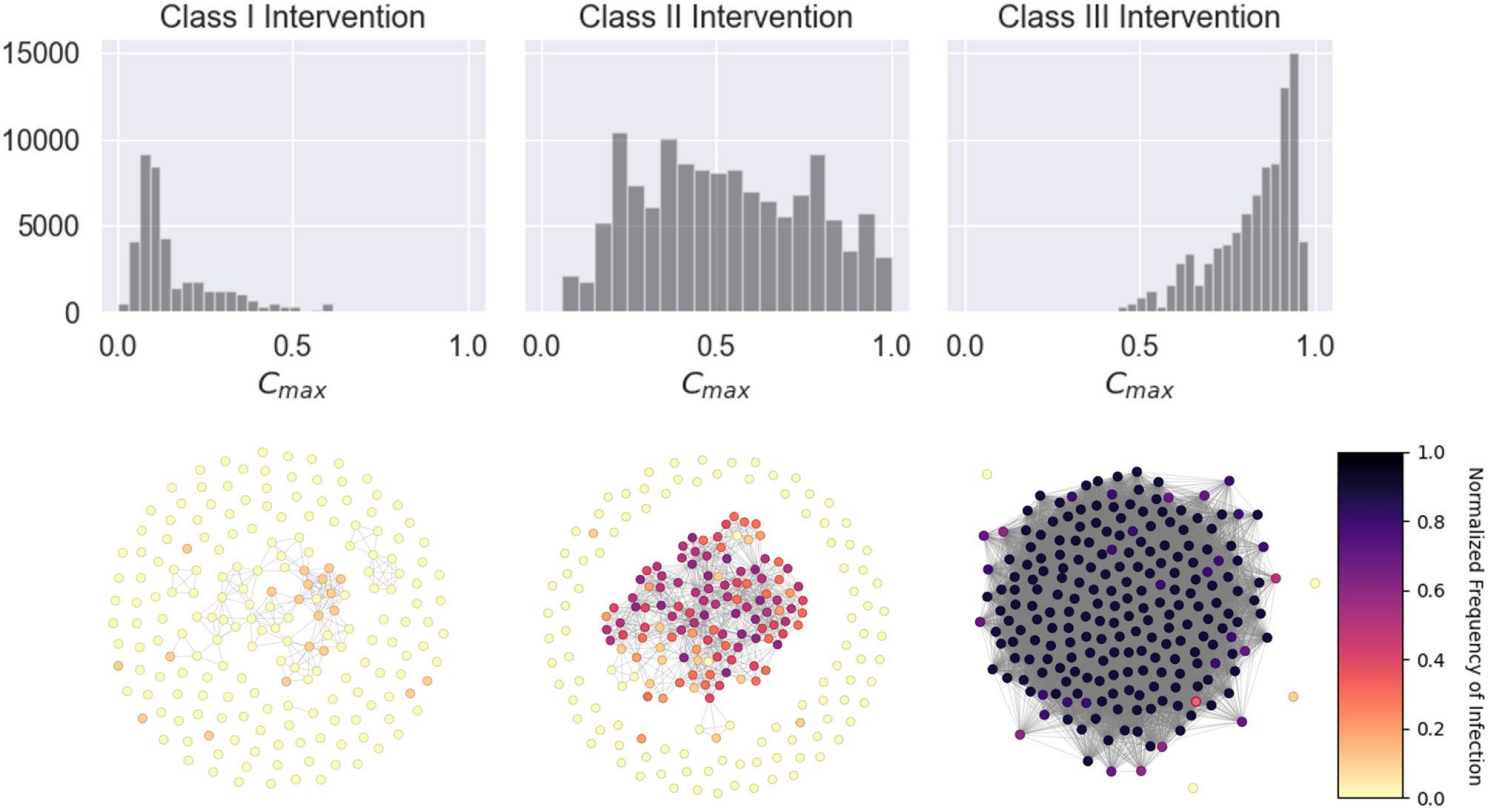
Maximum centrality distributions of contact networks for each intervention class, along with sample networks from all three classes. Colors of each node in the sample networks depict the normalized frequency of infection per node over 10 agent-based SIR simulations on the example network (darker colors represent higher risk).

Figure 9 displays $C_{\text {max}}$ distributions and example networks for all three classes of intervention. The normalized frequency of infection for each node in the example networks, over all simulations, is also shown. It can be seen that Class III networks tend to have hubs that are connected to nearly the entire network. This number is less for Class II and least for Class I. Furthermore, the example networks show how these hubs in Class III networks are nearly always likely to be infected, which in turn exposes the many nodes they are connected to (nearly half of the network). This effect is less in Class II, while in Class I the hubs have approximately the same (or even less) likelihood of infection in comparisons to other nodes.

**Figure 10:**
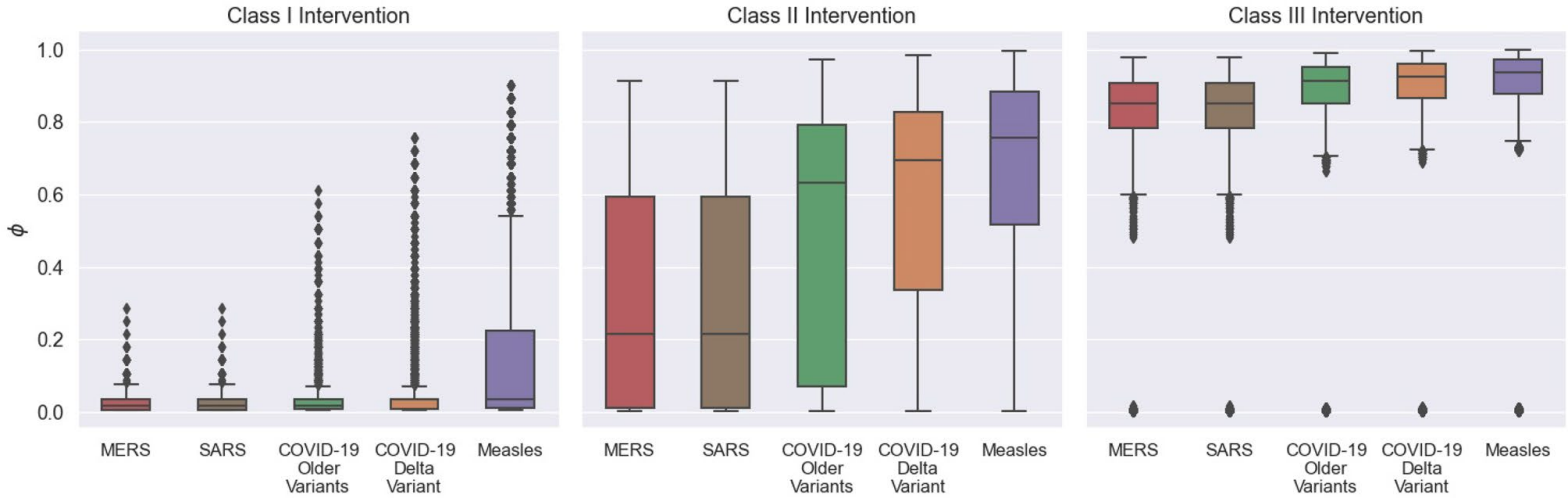
Effectiveness of Class I, II, and III strategies on outbreak size measured as final infected ratio, $\phi$, for different contagious respiratory viruses compared by secondary attack rate.

Finally, we compare the predicted efficacy of Class I, II, and III interventions on $\phi$ by relating $\rho$ to SAR for MERS, SARS, COVID-19 Delta and prior variants, and Measles. Figure 10 displays the predicted $\phi$ these diseases would have in the simulated environment under the three classes of intervention. The strictest Class I interventions are likely to prevent large outbreaks of MERS, SARS, and COVID-19, yet, Measles being an extremely transmissible disease, can still result in moderate outbreaks even under the strictest conditions. Class II interventions are likely to prevent MERS and SARS outbreaks, but are ineffective against Measles, and likely to allow significant outbreaks of COVID-19, likely infecting more that half the population. Class III interventions are likely to allow significantly large outbreaks of all four diseases with nearly the entire population at risk of infection. For each disease, Mann-Whitney U tests were performed to confirm whether there was a statistically significant improvement by having Class I interventions over Class II interventions ($H_0 : \text {Class I} \, \phi >= \text {Class II} \, \phi$) and Class II interventions over Class III interventions ($H_0 : \text {Class II} \, \phi >= \text {Class III} \, \phi$). The results for the Mann-Whitney U tests, shown in Table 2 show that for all considered diseases, Class I interventions were likely to have a significant reduction in final infected ratios over Class II interventions, as did Class II interventions over Class III interventions.

**Table 2:** Results from Mann-Whitney U tests for the null hypotheses $H_0 : {\text {Class I}} \, \phi >= {\text {Class II}} \, \phi$ and $H_0 : {\text {Class II}} \, \phi >= {\text {Class III}} \, \phi$.

Virus	$H_0 : {\text {Class I}} \, \phi >= {\text {Class II}} \, \phi$	$H_0 : {\text {Class II}} \, \phi >= {\text {Class III}} \, \phi$
MERS	($U=22359759.5$, $p~{\text {value}}=2.673\times 10^{-194}$)	($U=19540696.5$, $p~{\text {value}}<2.673\times 10^{-194}$)
SARS	($U=22359759.5$, $p~{\text {value}}=2.673\times 10^{-194}$)	($U=19540696.5$, $p~{\text {value}}<2.673\times 10^{-194}$)
COVID-19 Older Variants	($U=11010172.0$, $p~{\text {value}}<2.673\times 10^{-194}$)	($U=14979341.5$, $p~{\text {value}}<2.673\times 10^{-194}$)
COVID-19 Delta Variant	($U=7806042.0$, $p~{\text {value}}<2.673\times 10^{-194}$)	($U=17667952.5$, $p~{\text {value}}<2.673\times 10^{-194}$)
Measles	($U=26869646.0$, $p~{\text {value}}<2.673\times 10^{-194}$)	$U=202887512.0$, $p~{\text {value}}<2.673\times 10^{-194}$)

## Discussion

We present a simulation framework that couples a spatial agent-based model with an agent-based SIR model to evaluate the relative risk reduction of intervention strategies towards the prevention of outbreaks of contagious respiratory diseases such as COVID-19. The spatial agent-based model, implemented in AnyLogic, used blueprints and building data to provide a high-fidelity representation of the room and hallway allotments of the modeled space, including constraints such as dedicated entrances and exits, restrooms, break areas, and arrival schedules. The spatial agent-based model was run under varying non-pharmaceutical intervention strategies to generate contact networks. These interventions included movement restrictions (one-way vs unrestricted), hourly break probability, and population size (relative to current recommended capacity). The resulting contact networks were in turn utilized for simulations of the agent-based SIR model. Additionally, this two-stage modeling approach allowed us to cache the generated contact networks, which could then be used for further analysis through models other than the agent-based SIR model that we have used in this study. This is especially important as the higher fidelity of the spatial agent-based model demands more computational resources and time.

Our experiments provide insights into which intervention strategies are more successful at mitigating outbreaks and why. Firstly, for the tested floorplan, there is no significant reduction in risk by enforcing one-way movement (i.e. designating dedicated entrances or exits) over allowing for unrestricted movement. Instead, hourly break probability and population size have significant impacts on the risk of an outbreak, with the former having a much stronger effect. We confirm that this is due to the generation of contact networks with highly centralized hubs. The strongest impact on hub formation is seen with when individuals leave for breaks more often, followed by a lower, yet significant impact by population size.

We identify three classes of interventions based on the possible final outbreak sizes (final infected ratios) produced by these two parameters, over all possible degrees of transmissibility: Class I interventions with very strict restrictions on the frequency of leaving for breaks paired with very low population size, Class II interventions with a trade off between high (or low) break frequencies and low (or high) population size, and Class III interventions with both higher break frequencies and population size. We find that Class I restrictions lead to a significant decrease in outbreak size over Class II restrictions, for MERS, SARS, COVID-19, and Measles. Similarly, Class II restrictions lead to a significant decrease in outbreak size over Class III restrictions for all four viruses. Despite the advantage of Class I restrictions, it is important to consider the feasibility and ergonomic costs that such recommendations have on personnel. When the hourly likelihood of leaving one’s workstation is between 0.05 and 0.1, this translates to an overall probability between 0.34 and 0.57 that an individual would take at least one break during the whole 8 h work day. Although individuals could still take breaks inside their office spaces, this can lead to work-induced fatigue and isolation. In contrast, under Class II recommendations the hourly likelihood of taking a break is between 0.25 and 0.45, leading to a probability between 0.90 and 0.99 that individuals leave their office space at least once during the workday. However, Class II recommendations with higher hourly break likelihoods, say above 0.4 (translating to a 0.98 probability of at least one break in a workday), necessitate lower populations, in this case less than half of the recommended office capacity. In other words, despite the high efficacy of strict, Class I recommendations, a slightly more lenient Class II recommendation might be more ergonomic by restricting floor population below normal capacity, allowing individuals a more generous and safe movement to and from their designated workstations. However, Class II restrictions are still likely to result in significant COVID-19 outbreaks, especially considering the more contagious variants such as Delta and possibly Omicron, and must be combined with vaccination, which has not been modeled in this study, to lower transmission risk to a safe degree.

Our results concur with other studies that report a significant impact on the frequency of movement in comparison to other non-pharmaceutical interventions^24,32^. Break frequency as modeled in this study can be thought as the opposite of self-isolation, and many studies confirm the importance of self-isolation towards the reduction of epidemic size and duration^5,25,27,32^. However, targetted isolation based on the onset of symptoms can be less effective in the face of possible asymptomatic transmission^41–43^. In particular, a study of non-pharmaceutical interventions in a simulated grocery store environment finds that limiting simultaneous entries is more effective when population size is large^24^, which corroborates our finding that at large population sizes, the importance of break frequency is amplified.

This work provides a basis for future simulation studies that may benefit from the two-stage simulation approach presented in this paper. By modularizing the spatial and epidemiological aspects of contagious disease, we allow administrators and operations personnel to evaluate effects of spatial interventions independent of epidemiological model specifics made by computational epidemiologists. In other words, the spatial agent-based model may be thought of as a contact network generator for epidemiological models. As a result, it is important to note the significance of spatial layout towards the results presented in this study, as the contact networks generated are dependent on the degree of available paths of movement offered in the spatial agent-based model. Therefore, we do not claim that the relatively low importance of one-way movement restriction is generalizable to any floorplan, but rather, is limited to environments similar to the floorplan used in this study. Further experiments considering a range of commonly occurring floorplan geometries are required to provide a generalized estimation of the importance of movement restriction. Furthermore, in order to enable such testing, a system for automating the loading of blueprints to accommodate new floorplans would be valuable. Extensions to the current spatial model could also include factors such as air-flow and ventilation to produce airborne transmission networks. Additionally, as we are interested in the risks posed by the proposed interventions, we have not discriminated between changes in transmission rates under different forms of PPE usage or vaccination, which offers another future direction for this work. As the results we present with regards to the importance of the tested non-pharmaceutical interventions are based on the full range of transmission rate, [0, 1], it is likely that, for similar floorplans, the relative importance of the intervention parameters would remain the same, but that the final infected ratios by disease will likely be lower. Finally, although, we have considered CDC definitions of *prolonged contacts* in this study, minimal duration and distance of prolonged contacts can also be treated as parameters to generate the respective contact networks.

## Supplementary Information

Supplementary Information.

## Data Availability

AnyLogic spatial model file and SIR model source code have been made available at: https://github.com/chathika/spatial-SIR-COVID19-modeling.
